# Relationship between maternal pre‐pregnancy body mass index, gestational weight gain and childhood fatness at 6–7 years by air displacement plethysmography

**DOI:** 10.1111/mcn.12186

**Published:** 2015-04-08

**Authors:** Helen Castillo, Iná S. Santos, Alicia Matijasevich

**Affiliations:** ^1^ Department of Social Medicine Post Graduate Program of Epidemiology School of Medicine Federal University of Pelotas Pelotas Rio Grande do Sul Brazil; ^2^ Department of Preventive Medicine School of Medicine University of São Paulo São Paulo São Paulo Brazil

**Keywords:** body composition, pregnancy, children, fatness, women's weight, weight gain

## Abstract

This study aims to investigate the effect of maternal pre‐pregnancy body mass index (BMI) and gestational weight gain (GWG) on offspring body composition. In this prospective cohort study, offspring body composition at 6 years of age was obtained through air displacement plethysmography. Linear regression was used to obtain crude and adjusted coefficients. Information regarding offspring body composition and maternal pre‐pregnancy BMI was available for 3156 children and on offspring body composition and GWG for 3129 children. There was a direct association of maternal pre‐pregnancy BMI and GWG with offspring's fat mass (FM), fat‐free mass (FFM), fat mass index (FMI), fat‐free mass index (FFMI) and body fat percent (BF%) in crude and adjusted analyses. After adjustment for co‐variables, for each kg m^−2^ of maternal pre‐pregnancy BMI increase, there was a mean increment of 0.13 kg in the offspring FFM, 0.06 kg m^−2^ in FFMI, 0.11 kg in FM, 0.07 kg m^−2^ in FMI and 0.18% in BF%. For each kilogram of maternal GWG increase, there was a mean increment of 0.08 kg in offspring's FM, 0.05 kg m^−2^ in FMI, 0.04 kg in FFM, 0.01 kg m^−2^ in FFMI and 0.18 % in BF%. Mothers with a higher pre‐pregnancy BMI or GWG tend to have children with greater adiposity at age 6 years. Fetal overnutrition is more likely among mothers with greater BMI during pregnancy; as a consequence, it can accelerate the childhood obesity epidemic.

## Introduction

The increase in overweight and obesity prevalence observed in low‐ and middle ‐income countries has been recognised as an important public health problem (Adair *et al*. 2013). Maternal pre‐pregnancy body mass index (BMI) and/or excessive gestational weight gain (GWG) have been linked to maternal risks during pregnancy, including metabolic dysregulation and adverse outcomes for obstetrical care and delivery (Cedergren *et al*. 2008). Several observational studies assessed the association between maternal pre‐pregnancy obesity and increased risk of obesity in the offspring during childhood (Whitaker 2004; Mesman *et al*. 2009) and adulthood (Laitinen *et al*. 2001; Tequeanes *et al*. 2009). Some of the studies that investigated the role of maternal excessive GWG on obesity in the offspring suggested that excessive GWG might prenatally programme body fatness on childhood (Olson *et al*. 2009), adolescence (Oken *et al*. 2008) and adulthood (Mamun *et al*. 2009). Others found no effect of maternal GWG on obesity during childhood (Catalano *et al*. 1995) or adulthood (Koupil & Toivanen 2008).

Most of the studies conducted on children assessed the nutritional status through bioelectrical impedance and anthropometric measurements; however, these methods do not take into account the accumulation of fat‐free mass (FFM) during childhood growth (Wells & Fewtrell 2006). A handful of studies assessed body composition in neonates (Sewell *et al*. 2006; Hull *et al*. 2008, 2011; Au *et al*. 2013) and children (Burdette *et al*. 2006; Gale *et al*. 2007) through indirect methods, such as air displacement plethysmography (ADP), dual energy X‐ray absorptiometry (DXA), and body electrical conductivity (TOBEC); the investigators then evaluated the variability in the composition of FFM based on a two‐ or three‐compartment model (Wells & Fewtrell 2006). These studies reported a weak association between maternal pre‐pregnancy BMI and the increase in offspring obesity, whereas the effect of maternal GWG on offspring body composition remains unclear (Gale *et al*. 2007; Crozier *et al*. 2010; Hull *et al*. 2011). Therefore, the aim of the present study was to investigate the relationship between maternal pre‐pregnancy BMI and GWG on offspring fat mass (FM), fat mass index (FFI), body fat percent (BF%), fat‐free mass (FFM) and fat‐free mass index (FFMI) assessed by ADP using data from the 2004 Pelotas birth cohort study.

### Key messages

- Children of obese women before pregnancy have more adiposity at the age of 6 years than those of normal‐weight women.
- The greater the level of maternal education the greater the effect of maternal pre‐pregnancy BMI on offspring´s adiposity.
- Children of women who gained an excessive amount of weight during pregnancy have more adiposity at the age of 6 years than those of women who gained adequate amount of weight.

## Methods

In Pelotas city, Rio Grande do Sul State (Brazil), a third population‐based birth cohort study was initiated in 2004. All mothers who resided in the urban area of Pelotas or in the adjacent neighbourhood of Jardim América (part of the municipality of Capão do Leão) were approached for recruitment and interview within 24 h of delivery. All five maternity hospitals were visited daily and the mothers were interviewed via a standardised questionnaire by trained fieldworkers (perinatal study). Demographic, socio‐economic, reproductive, behavioural, prenatal care, gestational and pre‐gestational morbidities, and newborn characteristics were investigated (Santos *et al*. 2011). A total of 4231 live births (99.2% of all the eligible births) were included and followed‐up at 3 months and at 1, 2, 4 and 6 years of age, with 95.7%, 94.3%, 93.5%, 92.0% and 90.2% response rates, respectively. Current analysis used data collected at the perinatal study and the fifth follow‐up, when children were 6 years old (Santos *et al*. 2011, 2014).

### Maternal variables

The main exposure variables were maternal pre‐pregnancy BMI and GWG. Maternal pre‐pregnancy BMI was calculated as weight (kg) at the beginning of pregnancy divided by height in square meters (m^2^). Maternal weight at the beginning of pregnancy was extracted from the prenatal record or, when absent, by self‐report. Maternal height was measured in the household, at the first follow‐up visit, 3 months after delivery. BMI was classified into four categories: underweight (<18.5 kg m^−2^), normal weight (18.5–24.9 kg m^−2^), overweight (25.0–29.9 kg m^−2^) and obese (>30.0 kg m^−2^) (Institute of Medicine 2009).

Maternal GWG was calculated as the difference between pre‐pregnancy weight and the last recording of weight just before delivery. GWG was categorised according to the Institute of Medicine (IOM) guidelines as inadequate, adequate or excessive. Women in the underweight category should have a goal of 12.5–18.0 kg weight gain during pregnancy; those with normal BMI, 11.5–16.0 kg; overweight, 7.0–11.5 kg; and obese, 5.0–9.0 kg. Adequate weight gain for preterm and post‐term pregnancies was calculated using the expected weekly weight gain (Institute of Medicine 2009). Women below the lower limit were classified as inadequate; those within the range were classified as adequate; and those above the higher limit were classified as excessive GWG (Institute of Medicine 2009).

Other maternal covariates obtained from the perinatal study included family monthly income at birth (in quintiles), schooling at birth, skin colour (white, black, mulatto/brown, mixed or others), smoking and alcohol consumption during the pregnancy, parity, age and history of arterial hypertension and diabetes mellitus.

### Children's variables

Estimates of offspring body composition were obtained through ADP. The FMI and FFMI (in kg m^−2^) were calculated based on FM in kilograms or FFM in kilograms and height in square meters (m^2^). The indexed parameters have the advantage of taking into account the child height, which improves the sensitivity to detect changes in body composition measurements. Same‐height children can have different percentages of FM due to different absolute amounts of FM but equivalent amounts of FFM or different absolute amounts of FFM and same amounts of FM (Wells & Cole 2002). BF% was directly provided by ADP and corresponded to the total mass of fat divided by total body mass multiplied by 100. Weight was measured with a high precision scale with 0.01‐kg resolution (model BWB‐627‐A, Tanita, Tokyo, Japan). The Harpenden Stadiometer with 1 cm of precision was used for height measurements. Child sex and gestational age at birth (<37, 37–41 and >41 weeks) were collected at birth. Gestational age was estimated using an algorithm proposed by the National Center for Health Statistics (NCHS) based on the last menstrual period (Martin *et al*. 2005). If the birthweight, length and head circumference were inconsistent with the normal curves for the gestational age calculated, or if the date of the last menstrual period was unknown (Fenton 2003), then gestational age was determined using the Dubowitz method (Dubowitz *et al*. 1970), which was performed on almost all newborns.

### Statistical analyses

Analyses were carried out using Stata software, v. 12.0 (StataCorp, College Station, TX, USA). Descriptive statistics were calculated for all basic variables that compared children included in the analyses with those lost to follow‐up or with missing information on body composition and/or maternal pre‐pregnancy BMI and GWG. The chi‐square test and analysis of variance (ANOVA) assessed differences in characteristics between these groups. For comparison of offspring mean FM, FFM and BF%, according to maternal pre‐pregnancy BMI and GWG, the one‐way ANOVA test for homogeneous variances and the ANOVA test for linear trend were used. Linear regression was used to obtain crude and adjusted coefficients on offspring FM, FMI, FFM, FFMI and BF%. Adjusted analyses were conducted according to a hierarchical analytical model constructed in three levels. Family income, maternal schooling, maternal skin colour, maternal age, parity, pre‐gestational arterial hypertension, pre‐gestational diabetes and pre‐pregnancy BMI composed the first level. The second level comprised maternal characteristics (smoking and alcohol consumption during pregnancy, history of pre‐gestational arterial hypertension and diabetes mellitus, history of pregnancy‐induced arterial hypertension, history of gestational diabetes and maternal GWG) as well as child characteristics at birth (gestational age, weight and sex). In the third level, there were the outcomes of interest: offspring FM, FMI, FFM, FFMI or BF%, at 6 years of age. The effect of the maternal pre‐pregnancy BMI was adjusted for variables of the first level. The effect of GWG was controlled for variables of the first and second levels. Interaction between pre‐pregnancy BMI and maternal schooling over offspring FM, FMI and BF% was identified. The adjustment to the interaction term was included in the final multivariable model by using the MFPIgen command (a STATA command that permits to analyse all covariates as potential confounding factors), to select the confounder model at the 5% significance level and to identify interaction between two variables (Royston & Sauerbrei 2008). The regression models included a continuous‐by‐continuous interaction of the predictor variable with the interaction term (pre‐pregnancy BMI and maternal schooling).

The study protocol was approved by the Medical Ethics Committee of the Federal University of Pelotas. A written informed consent was obtained from the mothers before every follow‐up.

## Results

Information on offspring body composition and maternal pre‐pregnancy BMI was available for 3156 children, and on offspring's body composition and maternal GWG for 3129 children. Table 1 compares maternal and child characteristics of the children included in the analyses, excluding those with incomplete data. More than 70% of children from all the categories of the baseline characteristics were available for the current analyses. The exceptions were preterm and low‐birthweight children, those born to less educated mothers and those from families with low monthly income for whom the follow‐up rates were lower.

**Table 1:** Characteristics of mothers and children enrolled in the 2004 Pelotas birth cohort, and the percentage analysed according to the maternal exposures (Pelotas, Southern Brazil)

	Pregnancy BMI			GWG	
	*n* = 4231	*n* = 3156 (located)	*P*‐value	*n* = 3129 (located)	*P*‐value
Family income (quintiles)	–	–	<0.001	–	<0.001
1 (poorest)	872 (20.6)	578 (66.3)	–	568 (65.14)	–
2	855 (20.2)	638 (74.6)	–	631 (73.80)	–
3	816 (19.3)	641 (78.6)	–	639 (78.31)	–
4	858 (20.3)	681 (79.4)	–	676 (78.8)	–
5 (wealthiest)	830 (19.6)	618 (74.5)	–	615 (74.1)	–
Schooling (years)	–	–	<0.001	–	<0.001
Up to 4	655 (15.6)	424 (64.7)	–	419 (64.0)	–
5–8	1731 (41.3)	1305 (75.4)	–	1287 (74.4)	–
9–11	1382 (33.0)	1091 (78.9)	–	1088 (78.7)	–
≥12	420 (10.0)	309 (73.6)	–	308 (73.3)	–
Maternal skin colour	–	–	0.001	–	<0.001
White	2582 (61.7)	1982 (76.8)	–	1971 (76.3)	–
Black	689 (16.5)	495 (71.8)	–	488 (70.8)	–
Mulatto/Brown	869 (20.8)	614 (70.7)	–	605 (69.6)	–
Others	43 (1.0)	32 (74.4)	–	32 (74.4)	–
Maternal age (years)	–	–	0.36	–	0.5
<20	800 (18.9)	612 (76.5)	–	605 (75.6)	–
20–35	2987 (70.6)	2212 (74.1)	–	2195 (73.5)	–
>35	442 (10.5)	332 (75.1)	–	329 (74.4)	–
Primiparity	1666 (39.4)	1311 (78.7)	<0.001	1303 (78.2)	<0.001
Smoking (pregnancy)	1162 (27.5)	838 (72.1)	0.02	827 (71.2)	0.01
Child's sex	–	–	0.7	–	0.89
Boy	2196 (51.9)	1633 (74.4)	–	1622 (73.9)	–
Girl	2035 (48.1)	1523 (74.8)	–	1507 (74.1)	–
Gestational age (weeks)	–	–	<0.001	–	<0.001
<37	613 (14.5)	387 (63.1)	–	387 (61.7)	–
37–42	3468 (82.2)	2664 (76.8)	–	2562 (76.3)	–
>42	136 (3.2)	103 (75.7)	–	103 (75.7)	–
Birthweight (g)	–	–	<0.001	–	<0.001
<2500	424 (10.0)	237 (60.0)	–	231 (54.5)	–
2500–4000	3615 (85.5)	2773 (76.7)	–	2753 (76.2)	–
>4000	189 (4.5)	146 (77.3)	–	145 (76.7)	–

BMI, body mass index; GWG, gestational weight gain.

Table 2 shows the means of offspring FM, FFM, FMI, FFMI and BF% for maternal pre‐pregnancy BMI categorised into four groups, as well as for the maternal GWG categories and for GWG by maternal pre‐pregnancy BMI groups separately. There were direct associations between maternal pre‐pregnancy BMI and GWG with offspring FM, FFM, FMI, FFMI and BF%. The offspring FM, FFM, FMI, FFMI and BF% increased with the increase of maternal pre‐pregnancy BMI and GWG. Children from mothers with normal pre‐pregnancy BMI or overweight who gained excessive weight during pregnancy were found to have higher FM, FFM and FMI compared with children of mothers at the same pre‐pregnancy BMI who gained sufficient weight. Excessive maternal weight gain was associated with higher offspring FFMI only among children from mothers with normal pre‐pregnancy BMI.

**Table 2:** Fat mass, body fat percent and fat‐free mass means (and SD) by ADP at 6 years of age, according to maternal pre‐pregnancy BMI and across the categories of the 2009 Institute of Medicine's maternal gestational weight gain (GWG): 2004 cohort, 2010–2011 follow‐up (Pelotas, Southern Brazil)

Pre‐pregnancy BMI and GWG	Childhood body composition variables							
	*n*	Fat mass (kg)	Fat‐free mass (kg)	*n*	Fat mass index (kg m^−2^)	Fat‐free mass index (kg m^−2^)	*n*	Body fat percent (%)
		Mean (SD)	Mean (SD)		Mean (SD)	Mean (SD)		Mean (SD)
Pre‐pregnancy BMI	–	<0.001*	<0.001*	–	<0.001*	<0.001*	–	<0.001*
Underweight	139	4.5 (1.9)	17.3 (2.3)	137	3.1 (1.2)	12.1 (1.0)	139	20.1 (5.6)
Normal	1914	5.9 (3.2)	18.5 (2.7)	1890	4.0 (2.0)	12.6 (1.1)	1914	23.0 (7.5)
Overweight	751	6.9 (4.0)	19.3 (2.9)	747	4.5 (2.4)	12.8 (1.1)	751	24.7 (8.4)
Obese	352	8.0 (5.2)	19.6 (3.3)	349	5.3 (3.0)	13.2 (1.3)	352	26.8 (9.5)
Gestational weight gain	–	<0.001*	<0.001*	–	<0.001*	<0.001*	–	<0.001*
Insufficient	944	5.5 (3.1)	18.2 (2.7)	931	3.8 (2.0)	12.6 (1.1)	944	22.1 (7.4)
Sufficient	1142	6.1 (3.5)	18.7 (2.8)	1131	4.1 (2.1)	12.7 (1.1)	1142	23.5 (7.8)
Excessive	1043	7.2 (4.3)	19.3 (3.0)	1034	4.7 (2.5)	13.0 (1.2)	1043	25.4 (8.5)
GWG – underweight BMI	–	0.02*	0.20*	–	0.02	0.17	–	0.02*
Insufficient	48	3.8 (1.4)	17.0 (2.2)	47	2.8 (1.0)	11.8 (1.0)	48	18.4 (4.8)
Sufficient	73	4.8 (2.2)	17.6 (2.4)	72	3.3 (1.3)	12.2 (1.0)	73	21.0 (5.8)
Excessive	16	4.7 (1.5)	16.7 (2.0)	16	3.3 (1.0)	12.1 (0.8)	16	21.6 (5.5)
GWG – normal BMI	–	<0.001*	<0.001*	–	<0.001*	0.001	–	<0.001*
Insufficient	703	5.4 (3.0)	18.1 (2.6)	694	3.7 (1.8)	12.5 (1.1)	703	22.0 (7.3)
Sufficient	722	5.8 (3.2)	18.5 (2.8)	712	4.0 (2.0)	12.6 (1.1)	722	23.0 (7.4)
Excessive	473	6.7 (3.6)	19.1 (2.7)	468	4.4 (2.1)	12.8 (1.1)	473	24.6 (7.8)
GWG – overweight BMI	–	<0.001*	0.003*	–	0.002*	0.37	–	0.001*
Insufficient	121	5.8 (3.4)	18.6 (2.8)	120	4.0 (2.1)	12.8 (1.1)	121	22.4 (8.0)
Sufficient	227	6.6 (3.5)	19.0 (2.6)	227	4.4 (2.1)	12.8 (1.1)	227	24.4 (8.1)
Excessive	394	7.3 (4.3)	19.6 (3.0)	391	4.7 (2.5)	13.0 (1.2)	394	25.6 (8.5)
GWG – obese BMI	–	0.06*	0.37*	–	0.05*	0.32	–	0.06*
Insufficient	72	6.8 (4.0)	19.2 (2.7)	70	4.6 (2.5)	13.1 (1.1)	72	24.6 (8.7)
Sufficient	118	8.0 (5.0)	19.7 (3.4)	118	5.2 (3.0)	13.1 (1.3)	118	26.7 (9.5)
Excessive	159	8.5 (5.7)	19.8 (3.5)	158	5.6 (3.2)	13.3 (1.5)	159	27.8 (9.8)

BMI, body mass index; GWG, gestational weight gain. *P‐value from ANOVA for linear trend. Underweight: <18.5 kg m^−2^; Normal: 18.5–24.9; Overweight: 25.0–29.9; Obese: ≥30.0. GWG recommended ranges by maternal BMI: (1) <18, 5 kg m^−2^, 12.5–18 kg; (2) 18, 5–24, 9 kg m^−2^, 11.5–16 kg; (3) 25 and 29, 9 kg m^−2^, 11.5–16 kg, 7–11, 5 kg; and (4) ≥30, 0 kg m^−2^, 5–9 kg.

**Figure 1:**
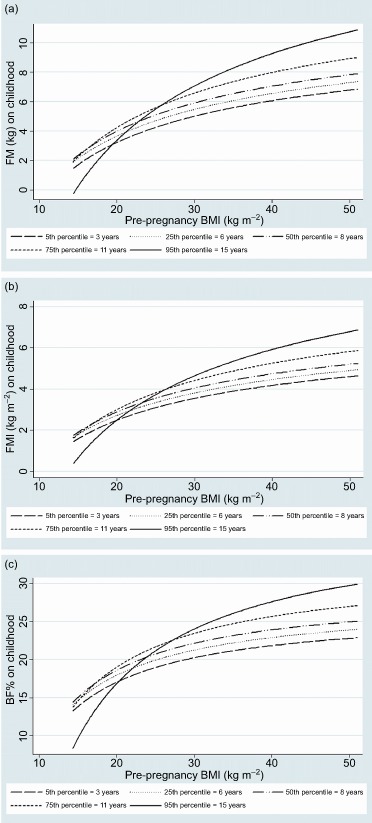
Adjusted linear predictor of fat mass (FM), fat mass index (FMI), body fat percent (BF%) and maternal pre‐pregnancy body mass index (BMI) according maternal schooling interaction term, determinate by ADP. 2004 cohort, 2010–2011 follow‐up (Pelotas, Southern Brazil). (a) Sliced plot of pre‐pregnancy BMI by maternal schooling; (b) sliced plot of pre‐pregnancy by maternal schooling; and (c) sliced plot of pre‐pregnancy BMI by maternal schooling.

Table 3 and Fig. 1 show results of the multivariable regression analyses for offspring body composition variables taking maternal pre‐pregnancy BMI and GWG as continuous variables. There was a direct association between maternal pre‐pregnancy BMI and GWG with offspring's FM, FFM, FMI, FFMI and BF% in crude and adjusted analyses. After adjustment for confounders, for each kg m^−2^ of maternal pre‐pregnancy BMI increase, there was a mean increment of 0.13 kg in the child's FFM and 0.06 kg m^−2^ in FFMI. For each kg of GWG increase, there was a mean increment of 0.08 kg in offspring's FM, 0.05 kg m^−2^ in FMI, 0.04 kg in FFM, 0.01 kg m^−2^ in FFMI and 0.18% in BF%. The greater the level of maternal education, the greater the effect of maternal pre‐pregnancy BMI on offspring's FM, FMI and BF%.

**Table 3:** Association between maternal pre‐pregnancy BMI, maternal gestational weight gain and childhood body composition estimated by ADP: 2004 cohort, 2010–2011 follow‐up (Pelotas, Southern Brazil)

Body composition	Linear regression coefficient and 95% CI									
	Pre‐pregnancy BMI (kg m^−2^)					Gestational weight gain (kg)				
	*n*	*β*	95% CI	*P*‐value	*R* ^2^	*n*	*β*	95% CI	*P*‐value	*R* ^2^
Fat‐free mass (kg)										
Crude	3156	0.12	(0.10, 0.14)	<0.001	3.69	3129	0.06	(0.04, 0.07)	<0.001	1.36
Adjust	3097	0.13*	(0.11, 0.15)	<0.001	11.39	3062	0.04^‡^	(0.02, 0.05)	<0.001	17.03
Fat‐free mass index (kg m^−2^)										
Crude	3123	0.05	(0.04, 0.06)	<0.001	3.96	3096	0.01	(0.006, 0.02)	<0.001	0.39
Adjust	3064	0.06*	(0.05, 0.07)	<0.001	8.67	3029	0.01^‡^	(0.006, 0.02)	0.001	11.51
Fat mass (kg)										
Crude	3156	0.2	(0.16, 0.22)	<0.001	5.1	3129	0.08	(0.06, 0.10)	<0.001	1.53
Adjust	3097	0.11^†^	(0.03, 0.18)	0.004	10.34	3062	0.08^‡^	(0.06, 0.11)	<0.001	12.82
Fat mass index (kg)										
Crude	3123	0.11	(0.10, 0.13)	<0.001	5.12	3096	0.04	(0.03, 0.06)	<0.001	1.29
Adjust	3064	0.07^†^	(0.02, 0.11)	0.003	10.06	3029	0.05^‡^	(0.04, 0.06)	<0.001	12.35
Body fat percent (%)										
Crude	3156	0.35	(0.30, 0.41)	<0.001	3.78	3129	0.16	(0.12, 0.21)	<0.001	1.43
Adjust	3097	0.18^†^	(0.03, 0.34)	0.02	11.39	3062	0.18^‡^	(0.13, 0.23)	<0.001	13.34

BMI, body mass index; CI, confidence interval; *R* ^2^, multiple linear regression analysis. *Adjust for family income, schooling at birth, skin colour, maternal age, parity and child's sex. ^†^Adjust for family income, schooling at birth, skin colour, maternal age, parity, child's sex and pre‐pregnancy BMI*schooling at birth. ^‡^Adjust for family income, schooling at birth, skin colour, maternal age, parity, maternal pre‐pregnancy BMI, maternal smoking and alcohol consumption during the pregnancy, history of arterial hypertension, history of diabetes mellitus, gestational age, birthweight and sex.

Maternal excessive weight gain was associated with greater offspring's FM, FMI, FFM and BF% (Table 4): taking maternal sufficient GWG as the category of reference (child's mean FM = 5.5 kg), there was a mean difference in FM of +1.01 kg (0.71, 1.32) among children from mothers with excessive GWG. The FMI of children from mothers who gained excessive weight was, on average, 0.57 kg m^−2^ (0.38, 0.75) greater than the FMI of children from mothers who gained sufficient weight (Table 4).

**Table 4:** Association between 2009 Institute of Medicine's maternal gestational weight gain (GWG) categories and body composition of children at 6 years of age estimated by ADP: cohort, 2010–2011 follow‐up (Pelotas, Southern Brazil)

	Linear regression coefficients									
	Crude					Adjust				
	*β*	95% CI	*P*‐value	*n*	*R* ^2^	*β*	95% CI	*P*‐value*	*n*	*R* ^2^
Fat‐free mass (kg)										
GWG Insufficient	−0.47	(−0.71, −0.22)	<0.001	3129	2.5	−0.11	(−0.34, 0.13)	0.37	362	17.01
GWG Sufficient	0	–	–	–	–	0	–	–	–	–
GWG Excessive	0.68	(0.44, 1.00)	<0.001	–	–	0.27	(0.04, 0.50)	0.02	–	–
Fat‐free mass index (kg m^−2^)										
GWG Insufficient	−0.12	(−0.23, 0.02)	0.02	3096	1.31	−0.05	(−0.15, 0.05)	0.32	3029	11.41
GWG Sufficient	0	–	–	–	–	0	–	–	–	–
GWG Excessive	0.21	(0.12, 0.31)	<0.001	–	–	0.07	(−0.02, 0.17)	0.14	–	–
Fat mass (kg)										
GWG Insufficient	−0.63	(−1.00, −0.32)	<0.001	3129	3.13	−0.28	(−0.60, 0.03)	0.08	3062	12.09
GWG Sufficient	0	–	–	–	–	0	–	–	–	–
GWG Excessive	1.01	(0.71, 1.32)	<0.001	–	–	0.6	(0.30, 0.90)	<0.001	–	–
Fat mass index (kg m^−2^)										
GWG Insufficient	−0.37	(−0.56, −0.18)	<0.001	3096	2.8	−0.18	(−0.37, 0.003)	0.05	3029	11.52
GWG Sufficient	0	–	–	–	–	0	–	–	–	–
GWG Excessive	0.57	(0.38, 0.75)	<0.001	–	–	0.34	(0.16, 0.52)	<0.001	–	–
Body fat percent (%)										
GWG Insufficient	−1.38	(−2.06, −0.70)	<0.001	3129	2.67	−0.72	(−1.40, −0.05)	0.04	3062	12.57
GWG Sufficient	0	–	–	–	–	0	–	–	–	–
GWG Excessive	2.41	(1.61, 3.20)	<0.001	–	–	1.25	(0.60, 1.90)	<0.001	–	–

CI, confidence interval. *Adjust for family income, schooling at birth, skin colour, maternal age, parity, maternal pre‐pregnancy body mass index, maternal smoking and alcohol consumption during the pregnancy, history of arterial hypertension, history of diabetes mellitus, gestational age, weight at birth and child's sex (*P*‐value and *R* ^2^ of multiple linear regression analysis). *P*‐value of testparm ≤0.001 in all crude analyses.

## Discussion

In this prospective study, maternal pre‐pregnancy BMI and GWG were associated with FM, FMI, FFM and BF% in offspring at 6 years of age. The linear multivariable regression models showed that offspring's FM, FMI, FFM, FFMI and BF% were more strongly associated with maternal pre‐pregnancy BMI than with GWG.

The children born to overweight or obese mothers before pregnancy presented higher amount of FM than children born to normal or underweight mothers. Two previous studies showed a positive association of maternal pre‐pregnancy BMI with FM in childhood (Berkowitz *et al*. 2005; Lawlor *et al*. 2008) and one in infancy (Sewell *et al*. 2006). The effect size of maternal pre‐pregnancy BMI on offspring's FM in the present study (*β* = 0.22 kg) was similar to that reported by Berkowitz *et al*. (2005) (*β* = 0.27 kg) at 6 years of age and by Lawlor *et al*. (2008) (*β* = 0.25 kg) among children aged 9–11 years. Similarly, with regard to BF%, the present results are in agreement with the reports of three studies, two of infants (Sewell *et al*. 2006; Hull *et al*. 2008) and one with 6‐year‐old children (Berkowitz *et al*. 2005).

A prospective study by Gale *et al*. (2007) showed a direct association between pre‐pregnancy BMI and FMI after stratifying by sex. The present study found the same direct association but no differences by sex. Children of Gale's study were 9 years old and had probably passed the timing of adiposity rebound; this can explain the greater fatness observed in girls compared with boys. Similarly, with univariate analysis, the association between maternal pre‐pregnancy BMI and offspring FFM was observed by Hull *et al*. (2008) in neonates and by Berkowitz *et al*. (2005) in 4‐ and 6‐year‐old children. In a study by Lawlor *et al*. (2008), the regression coefficients in crude (*β* = 0.09 kg) and adjusted (*β* = 0.10 kg) analyses were identical to the results (*β* = 0.10 kg) of the present study. The correlation between maternal pre‐pregnancy BMI and offspring FFMI is different from the results reported by Gale *et al*. (2007) that did not find any association.

In the current study, a positive interaction was found between pre‐pregnancy BMI and maternal education with regard to childhood obesity. Offspring's FM, FMI and BF% were particularly higher among those from obese mothers with 15 years of formal education. The authors did not find any other study in the literature that reported such an interaction. No interaction was observed between pre‐pregnancy BMI and maternal education over offspring's FFM and FFMI. Previous studies that investigated the effect of maternal education on offspring's body composition found negative association (Schnurbein *et al*. 2011), lack of association (Burdette *et al*. 2006) or positive association with offspring adiposity (Leary *et al*. 2006; Zanini *et al*. 2014).

In this study, a direct relationship between maternal GWG and offspring's body composition indicators was found. Hull *et al*. (2011) reported an interaction between GWG and pre‐pregnancy BMI (greater adiposity among children from overweight mothers with excessive GWG compared with children from overweight mothers with sufficient GWG). In the current study, no interaction between GWG and pre‐pregnancy BMI was found.

Methodological issues may explain at least part of the differences observed in the study results. All of the published studies were conducted in high‐income countries as the United States (Berkowitz *et al*. 2005; Burdette *et al*. 2006; Sewell *et al*. 2006; Hull *et al*. 2008, 2011), the United Kingdom (Gale *et al*. 2007; Lawlor *et al*. 2008; Crozier *et al*. 2010) and Australia (Au *et al*. 2013). To the authors' knowledge, this is the first analysis of such associations in a middle‐income country. The Pelotas 2004 birth cohort has the advantage of including a large sample of children with maternal anthropometric measurements (pre‐pregnancy BMI and maternal GWG), whereas most of the studies were composed of smaller samples (Berkowitz *et al*. 2005; Burdette *et al*. 2006; Sewell *et al*. 2006; Hull *et al*. 2008, 2011; Crozier *et al*. 2010). Except three studies (Burdette *et al*. 2006; Sewell *et al*. 2006; Au *et al*. 2013), all others selected only Caucasian participants. Our study included a mixed sample, as is the ethnic profile of the Brazilian population. Most of the studies analysed maternal overweight and/or obesity as a categorical variable. Our study explored the effect of pre‐pregnancy BMI and GWG as continuous variables. According to the offspring age, when body composition was assessed, some studies included newborns (Sewell *et al*. 2006; Hull *et al*. 2008, 2011; Au *et al*. 2013), and others, children aged 4–11 years old. Also, the methods used for assessment of body composition included DXA in four studies (Berkowitz *et al*. 2005; Burdette *et al*. 2006; Gale *et al*. 2007; Lawlor *et al*. 2008), ADP in three (Hull *et al*. 2008, 2011; Au *et al*. 2013) and TOBEC in one (Sewell *et al*. 2006).

This study has strengths and limitations. The principal strength is the high follow‐up rate (90.2%) of the cohort participants at 6 years of age, thus allowing the extrapolation of the present results to the population of Pelotas as well as to other populations with similar economic and socio‐cultural contexts. Anthropometric measurements were carried out with standard methodology and trained fieldworkers performed the equipment management. Also, one of the major strengths of the study was that children's body composition was measured using a reference method. A limitation of the study was the use of maternal self‐reported weight before pregnancy or the record of weight at the first prenatal visit, which can lead to some degree of classification error in the exposures. Although self‐reported maternal weight has a high correlation with weight measurement (Stevens‐Simon *et al*. 1992), women generally underestimate their own weight (Yu & Nagey 1992). In addition, gestational age at the beginning of prenatal care can vary among women. However, self‐reported pre‐pregnancy weight and measured weight at first prenatal visit resulted in an identical category classification of pre‐pregnancy BMI (Holland *et al*. 2013). Additionaly, 98% of the mothers from the Pelotas 2004 cohort attended antenatal care, most of them (72.3%) starting at the first trimester of pregnancy (Victora *et al*. 2008).

Maternal gestational as well as pre‐gestational diabetes mellitus is one of the known risk factors for offspring adiposity and is also associated with maternal obesity. According to the intrauterine overnutrition hypothesis, high maternal plasma concentrations of glucose, free fatty acids and amino acids are more likely among mothers with greater BMI during pregnancy, and consequently, fetal overnutrition (Burdette *et al*. 2006; Armitage *et al*. 2008). We conducted sensitivity analyses excluding this group of mothers, but no difference was observed in the coefficients, possibly due to the small number of mothers with diabetes in our sample (*n* = 13).

## Conclusion

The impact of maternal BMI and GWG on offspring's body composition was demonstrated through associations with linear trends, which strengthens the hypothesis of a causal relationship based on the biological gradient observed. The results of this study increase the understanding of the direct effect of maternal obesity on the developing child, the fetal overnutrition hypothesis, and the trans‐generational cycle of obesity.

Data from the three population‐based birth cohorts in Pelotas show that the mean pre‐pregnancy BMI increased from 22.7 kg m^−2^ in 1982 to 22.8 in 1993, and then to 24.2 in 2004 (Victora *et al*. 2008). There was a marked increase in maternal pre‐pregnancy obesity prevalence during the 22‐year period (from 4.4% in 1982 to 4.9% in 1993, and then 10.7% in 2004) (Victora *et al*. 2008). In addition, compared with mothers during the time points of 1982 and 1993, the 2004 mothers gained an average of 660 g more than the 1982 mothers and 800 g more than the 1993 mothers during pregnancy (Victora *et al*. 2008). In such a scenario, effective strategies for prevention of the trans‐generational cycle of obesity are limited. Women should not only be within a normal BMI range when they conceive but should also gain within the ranges recommended by the IOM guidelines. Although the effect of maternal pre‐pregnancy BMI on the child adiposity is stronger than the effect of GWG, antenatal care may represent a more viable time period to interrupt this cycle, at least in low‐ and middle‐income settings where maternal obesity prevalence is higher. Health care providers in those settings need to routinely classify maternal BMI at the beginning of pregnancy and to advise mothers to maintain their weight gain within the recommended ranges.

## Source of funding

This article is based on data from the study ‘Pelotas Birth Cohort, 2004’ conducted by Postgraduate Program in Epidemiology at Universidade Federal de Pelotas, with the collaboration of the Brazilian Public Health Association (ABRASCO). From 2009 to 2013, the Wellcome Trust supported the 2004 birth cohort study. The World Health Organization, National Support Program for Centers of Excellence (PRONEX), Brazilian National Research Council (CNPq), Brazilian Ministry of Health, and Children's Pastorate supported previous phases of the study. I. S. Santos and A. Matijasevich receive research support from the National Council for Scientific and Technological Development (CNPq), Brazil.

## Conflicts of interest

The authors declare that they have no conflicts of interest.

## Contributions

HC and ISS conceived the paper, conducted the analysis and wrote the manuscript. AM contributed to the design of the study. All authors revised and approved the final version of the paper for publication.

## References

[mcn12186-bib-0001] Adair L.S. , Fall C.H. , Osmond C. , Stein A.D. , Martorell R. , Ramirez‐Zea M. *et al* (2013) Associations of linear growth and relative weight gain during early life with adult health and human capital in countries of low and middle income: findings from five birth cohort studies. Lancet 382 (9891), 525–534. 23541370 10.1016/S0140-6736(13)60103-8 PMC3744751

[mcn12186-bib-0002] Armitage J.A. , Poston L. & Taylor P.D. (2008) Developmental origins of obesity and the metabolic syndrome: the role of maternal obesity. Frontiers of Hormone Research 36, 73–84. 18230895 10.1159/000115355

[mcn12186-bib-0003] Au C.P. , Raynes‐Greenow C.H. , Turner R.M. , Carberry A.E. & Jeffery H. (2013) Fetal and maternal factors associated with neonatal adiposity as measured by air displacement plethysmography: a large cross‐sectional study. Early Human Development 89 (10), 839–843. 23968962 10.1016/j.earlhumdev.2013.07.028

[mcn12186-bib-0004] Berkowitz R.I. , Stallings V.A. , Maislin G. & Stunkard A.J. (2005) Growth of children at high risk of obesity during the first 6 years of life: implications for prevention. The American Journal of Clinical Nutrition 81 (1), 140–146. 15640473 10.1093/ajcn/81.1.140

[mcn12186-bib-0005] Burdette H.L. , Whitaker R.C. , Hall W.C. & Daniels S.R. (2006) Maternal infant‐feeding style and children's adiposity at 5 years of age. Archives of Pediatrics and Adolescent Medicine 160 (5), 513–520. 16651495 10.1001/archpedi.160.5.513

[mcn12186-bib-0006] Catalano P.M. , Drago N.M. & Amini S.B. (1995) Maternal carbohydrate metabolism and its relationship to fetal growth and body composition. American Journal of Obstetrics & Gynecology 172 (5), 1464–1470. 7755055 10.1016/0002-9378(95)90479-4

[mcn12186-bib-0007] Cedergren M. , Brynhildsen J. , Josefsson A. , Sydsjo A. & Sydsjo G. (2008) Hyperemesis gravidarum that requires hospitalization and the use of antiemetic drugs in relation to maternal body composition. American Journal of Obstetrics & Gynecology 198 (4), 411–415. 10.1016/j.ajog.2007.09.029 18221931

[mcn12186-bib-0008] Crozier S.R. , Inskip H.M. , Godfrey K.M. , Cooper C. , Harvey N.C. , Cole Z.A. *et al* (2010) Weight gain in pregnancy and childhood body composition: findings from the Southampton Women's Survey. The American Journal of Clinical Nutrition 91 (6), 1745–1751. 20375187 10.3945/ajcn.2009.29128 PMC3091013

[mcn12186-bib-0009] Dubowitz L.M. , Dubowitz V. & Goldberg C. (1970) Clinical assessment of gestational age in the newborn infant. Journal of Pediatrics 77 (1), 1–10. 5430794 10.1016/s0022-3476(70)80038-5

[mcn12186-bib-0010] Fenton T.R. (2003) A new growth chart for preterm babies: Babson and Benda's chart updated with recent data and a new format. BMC Pediatrics 13 (3), 1471–2431. 10.1186/1471-2431-3-13 PMC324406 14678563

[mcn12186-bib-0011] Gale C.R. , Javaid M.K. , Robinson S.M. , Law C.M. , Godfrey K.M. & Cooper C. (2007) Maternal size in pregnancy and body composition in children. The Journal of Clinical Endocrinology and Metabolism 92 (10), 3904–3911. 17684051 10.1210/jc.2007-0088 PMC2066182

[mcn12186-bib-0012] Holland E. , Moore Simas T.A. , Doyle Curiale D.K. , Liao X. & Waring M.E. (2013) Self‐reported pre‐pregnancy weight vs. weight measured at first prenatal visit: effects on categorization of pre‐pregnancy body mass index. Maternal and Child Health Journal 17 (10), 1872–1878. 23247668 10.1007/s10995-012-1210-9 PMC3622142

[mcn12186-bib-0013] Hull H.R. , Dinger M.K. , Knehans A.W. , Thompson D.M. & Fields D.A. (2008) Impact of maternal body mass index on neonate birthweight and body composition. American Journal of Obstetrics & Gynecology 198 (4), 411–416. 10.1016/j.ajog.2007.10.796 18279830

[mcn12186-bib-0014] Hull H.R. , Thornton J.C. , Ji Y. , Paley C. , Rosenn B. , Mathews P. *et al* (2011) Higher infant body fat with excessive gestational weight gain in overweight women. American Journal of Obstetrics & Gynecology 205 (3), 211–217. 21621185 10.1016/j.ajog.2011.04.004 PMC3170486

[mcn12186-bib-0015] Institute of Medicine (2009) Weight Gain During Pregnancy: Reexamining the Guidelines. K.M. Rasmussen & A.L. Yaktine: Washington, DC. 20669500

[mcn12186-bib-0016] Koupil I. & Toivanen P. (2008) Social and early‐life determinants of overweight and obesity in 18‐year‐old Swedish men. International Journal of Obesity 32 (1), 73–81. 17667914 10.1038/sj.ijo.0803681

[mcn12186-bib-0017] Laitinen J. , Power C. & Jarvelin M.R. (2001) Family social class, maternal body mass index, childhood body mass index, and age at menarche as predictors of adult obesity. The American Journal of Clinical Nutrition 74 (3), 287–294. 11522550 10.1093/ajcn/74.3.287

[mcn12186-bib-0018] Lawlor D.A. , Timpson N.J. , Harbord R.M. , Leary S. , Ness A. , McCarthy M.I. *et al* (2008) Exploring the developmental overnutrition hypothesis using parental‐offspring associations and FTO as an instrumental variable. PLoS Medicine 5 (3), 484–493. 10.1371/journal.pmed.0050033 PMC2265763 18336062

[mcn12186-bib-0019] Leary S.D. , Smith G.D. , Rogers I.S. , Reilly J.J. , Wells J.C. & Ness A.R. (2006) Smoking during pregnancy and offspring fat and lean mass in childhood. Obesity 14, 2284–2293. 17189557 10.1038/oby.2006.268 PMC1890311

[mcn12186-bib-0020] Mamun A.A. , O'Callaghan M. , Callaway L. , Williams G. , Najman J. & Lawlor D.A. (2009) Associations of gestational weight gain with offspring body mass index and blood pressure at 21 years of age: evidence from a birth cohort study. Circulation 119 (13), 1720–1727. 19307476 10.1161/CIRCULATIONAHA.108.813436

[mcn12186-bib-0021] Martin J.A. , Hamilton B.E. , Sutton P.D. , Ventura S.J. , Menacker F. & Munson M.L. (2005) Births: final data for 2003. National Vital Statistics Reports 54 (2), 1–116. 16176060

[mcn12186-bib-0022] Mesman I. , Roseboom T.J. , Bonsel G.J. , Gemke R.J. , van der Wal M.F. & Vrijkotte T.G. (2009) Maternal pre‐pregnancy body mass index explains infant's weight and BMI at 14 months: results from a multi‐ethnic birth cohort study. Archives of Disease in Childhood 94 (8), 587–595. 19332418 10.1136/adc.2008.137737

[mcn12186-bib-0023] Oken E. , Rifas‐Shiman S.L. , Field A.E. , Frazier A.L. & Gillman M.W. (2008) Maternal gestational weight gain and offspring weight in adolescence. Obstetrics & Gynecology 112 (5), 999–1006. 18978098 10.1097/AOG.0b013e31818a5d50 PMC3001295

[mcn12186-bib-0024] Olson C.M. , Strawderman M.S. & Dennison B.A. (2009) Maternal weight gain during pregnancy and child weight at age 3 years. Maternal and Child Health Journal 13 (6), 839–846. 18818995 10.1007/s10995-008-0413-6

[mcn12186-bib-0025] Royston P. & Sauerbrei W. (2008) Interactions. *Multivariable Model‐Building: a pragmatic approach to regression analysis based on fractional polynomials for modelling continuous variables* 151–182.

[mcn12186-bib-0026] Santos I.S. , Barros A.J. , Matijasevich A. , Domingues M.R. , Barros F.C. & Victora C.G. (2011) Cohort profile: the 2004 Pelotas (Brazil) birth cohort study. International Journal of Epidemiology 40 (6), 1461–1468. 20702597 10.1093/ije/dyq130 PMC3235016

[mcn12186-bib-0027] Santos I.S. , Barros A.J. , Matijasevich A. , Zanini R. , Chrestani Cesar M.A. , Camargo F.A. *et al* (2014) Cohort profile update: 2004 Pelotas (Brazil) Birth Cohort Study. Body composition, mental health 5 and genetic assessment at the 6 years follow‐up. International Journal of Epidemiology 43 (5), 1437–1437f. 25063002 10.1093/ije/dyu144 PMC4190519

[mcn12186-bib-0028] Schnurbein J. , Klenk J. , Galm C. , Berg S. , Gottmann P. , Steinacker J.M. *et al* (2011) Reference values and early determinants of intra‐abdominal fat mass in primary school children. Hormone Research in Paediatrics 75, 412–422. 21335951 10.1159/000324110

[mcn12186-bib-0029] Sewell M.F. , Huston‐Presley L. , Super D.M. & Catalano P. (2006) Increased neonatal fat mass, not lean body mass, is associated with maternal obesity. American Journal of Obstetrics & Gynecology 195 (4), 1100–1103. 16875645 10.1016/j.ajog.2006.06.014

[mcn12186-bib-0030] Stevens‐Simon C. , Roghmann K.J. & McAnarney E.R. (1992) Relationship of self‐reported prepregnant weight and weight gain during pregnancy to maternal body habitus and age. Journal of the American Dietetic Association 92 (1), 85–87. 1728630

[mcn12186-bib-0031] Tequeanes A.L. , Gigante D.P. , Assuncao M.C. , Chica D.A. & Horta B.L. (2009) Maternal anthropometry is associated with the body mass index and waist : height ratio of offspring at 23 years of age. Journal of Nutrition 139 (4), 750–754. 19211832 10.3945/jn.108.100669

[mcn12186-bib-0032] Victora C.G. , Matijasevich A. , Santos I.S. , Barros A.J.D. , Horta B.L. & Barros F.C. (2008) Breastfeeding and feeding patterns in three birth cohorts in Southern Brazil: trends and differentials. Cadernos de Saúde Pública 24, 409–416. 18797716 10.1590/s0102-311x2008001500006 PMC3532951

[mcn12186-bib-0033] Wells J.C. & Cole T.J. (2002) Adjustment of fat‐free mass and fat mass for height in children aged 8 years. International Journal of Obesity and Related Metabolic Disorders 26 (7), 947–952. 12080448 10.1038/sj.ijo.0802027

[mcn12186-bib-0034] Wells J.C. & Fewtrell M.S. (2006) Measuring body composition. Archives of Disease in Childhood 91 (7), 612–617. 16790722 10.1136/adc.2005.085522 PMC2082845

[mcn12186-bib-0035] Whitaker R.C. (2004) Predicting preschooler obesity at birth: the role of maternal obesity in early pregnancy. Pediatrics 114 (1), 29–36. 10.1542/peds.114.1.e29 15231970

[mcn12186-bib-0036] Yu S.M. & Nagey D.A. (1992) Validity of self‐reported pregravid weight. Annals of Epidemiology 2 (5), 715–721. 1342323 10.1016/1047-2797(92)90016-j

[mcn12186-bib-0037] Zanini R.V. , Santos I.S. , Gigante D.P. , Matijasevich A. , Fernando C. , Barros F.C. *et al* (2014) Body composition assessment using DXA in six‐year‐old children: the 2004 Pelotas Birth Cohort, Rio Grande do Sul State, Brazil. Cadernos de Saúde Pública 30 (10), 2123–2133. 25388315 10.1590/0102-311x00153313

